# The gut microbiome in pancreatogenic diabetes differs from that of Type 1 and Type 2 diabetes

**DOI:** 10.1038/s41598-021-90024-w

**Published:** 2021-05-26

**Authors:** Rupjyoti Talukdar, Priyanka Sarkar, Aparna Jakkampudi, Subhaleena Sarkar, Mohsin Aslam, Manasa Jandhyala, G. Deepika, Misbah Unnisa, D. Nageshwar Reddy

**Affiliations:** 1 Pancreas Research Group and Division of Gut Microbiome Research, Wellcome DBT India Alliance Laboratories, Institute of Basic and Translational Research, Asian Healthcare Foundation, Asian Institute of Gastroenterology, Hyderabad, Telangana 500032 India; 2 Department of Medical Gastroenterology, Asian Institute of Gastroenterology, Hyderabad, India; 3 Department of Endocrinology, Asian Institute of Gastroenterology, Hyderabad, India; 4 Department of Biochemistry, Asian Institute of Gastroenterology, Hyderabad, India

**Keywords:** Chronic pancreatitis, Diabetes

## Abstract

We hypothesized that the gut microbiome in patients with diabetes secondary to chronic pancreatitis (Type 3c) is different from those with Type 1 and Type 2 diabetes. This was a cross-sectional preliminary study that included 8 patients with Type 1, 10 with Type 2, 17 with Type 3c diabetes and 9 healthy controls. Demographic, clinical, biochemical, imaging and treatment data were recorded and sequencing of the V3–V4 region of the bacterial 16SrRNA was done on fecal samples. Bioinformatics and statistical analyses was performed to evaluate the differences in the diversity indices, distance matrices, relative abundances and uniqueness of organisms between the types of diabetes. There was significant difference in the species richness. Beta diversity was significantly different between patients with Type 3c diabetes and the other groups. 31 genera were common to all the three types of diabetes. There was significant differences in the species level taxa between Type 3c diabetes and the other groups. The unique bacterial species signature in Type 3c diabetes compared to Type 1 and Type 2 diabetes included *Nesterenkonia* sp.* AN1, Clostridium magnum, Acinetobacter lwoffii, Clostridium septicum, Porphyromonas somerae, Terrabacter tumescens, and Synechococus* sp.

## Introduction

India harbours 77 million diabetics, which amounts to a national prevalence of 8.9%^1^. While Type 2 diabetes is the most common form of diabetes, the prevalence of Type 1 DM is also on the rise in India at a rate of 3–5% every year^2^. Likewise, there is also a substantially high frequency (~ 38%) of Type 3c diabetes in India^3^. Type 3c diabetes is defined as diabetes secondary to disease of the exocrine pancreas^4^. The frequency of Type 3c diabetes among patients with CP in India ranges from around 17% in early onset CP to over 60% in late onset CP^5–8^. Moreover, the age of onset of this condition is generally lower in India compared to that in the west^6,7^.

It is now clear that the normal gut microbial ecology has a substantial role in maintaining health through optimal nutrient digestion, maintenance of gastrointestinal function, synthesis of vitamins and pro-health metabolites, metabolism of drugs and xenobiotics, and glycemic and lipidomic control^9–12^. There is now substantial evidence from the west and a handful from India that there is significant gut microbial dysbiosis in Type 1 and Type 2 diabetes^13–19^. In our recent study on gut microbial dysbiosis in CP, we observed alteration of the microbiome even in patients with Type 3c diabetes^20^. From a mechanistic perspective, the pathogenesis of the three types of diabetes differs profoundly. While Type 1 and Type 2 diabetes are associated with insulin deficiency resulting from autoimmunity and insulin resistance respectively, Type 3c diabetes shows a combination of insulin deficiency and hepatic insulin resistance^4,21–25^. Further, current evidence suggests infiltration of Th17 cells into the islets in CP that secretes IFN-γ resulting in a functional defect in the beta-cells^26–28^. There is also a loss of pancreatic polypeptide response that results in hepatic insulin resistance in Type 3c diabetes^3^.

On the premise that the pathophysiology of Type 3c diabetes differ from that of Type 1 and Type 2 diabetes, we hypothesized that the gut microbiome in Type 3c diabetes will also differ from Type 1 and Type 2 diabetes. Even though gut microbial dysbiosis has been reported in all these three types of diabetes separately, no head to head comparison have yet been evaluated. We therefore embarked on the current study wherein the objective was to evaluate any difference in the gut microbiome between the three types of diabetes.

## Results

### Patients and fecal metagenomic characteristics

**Figure 1:**
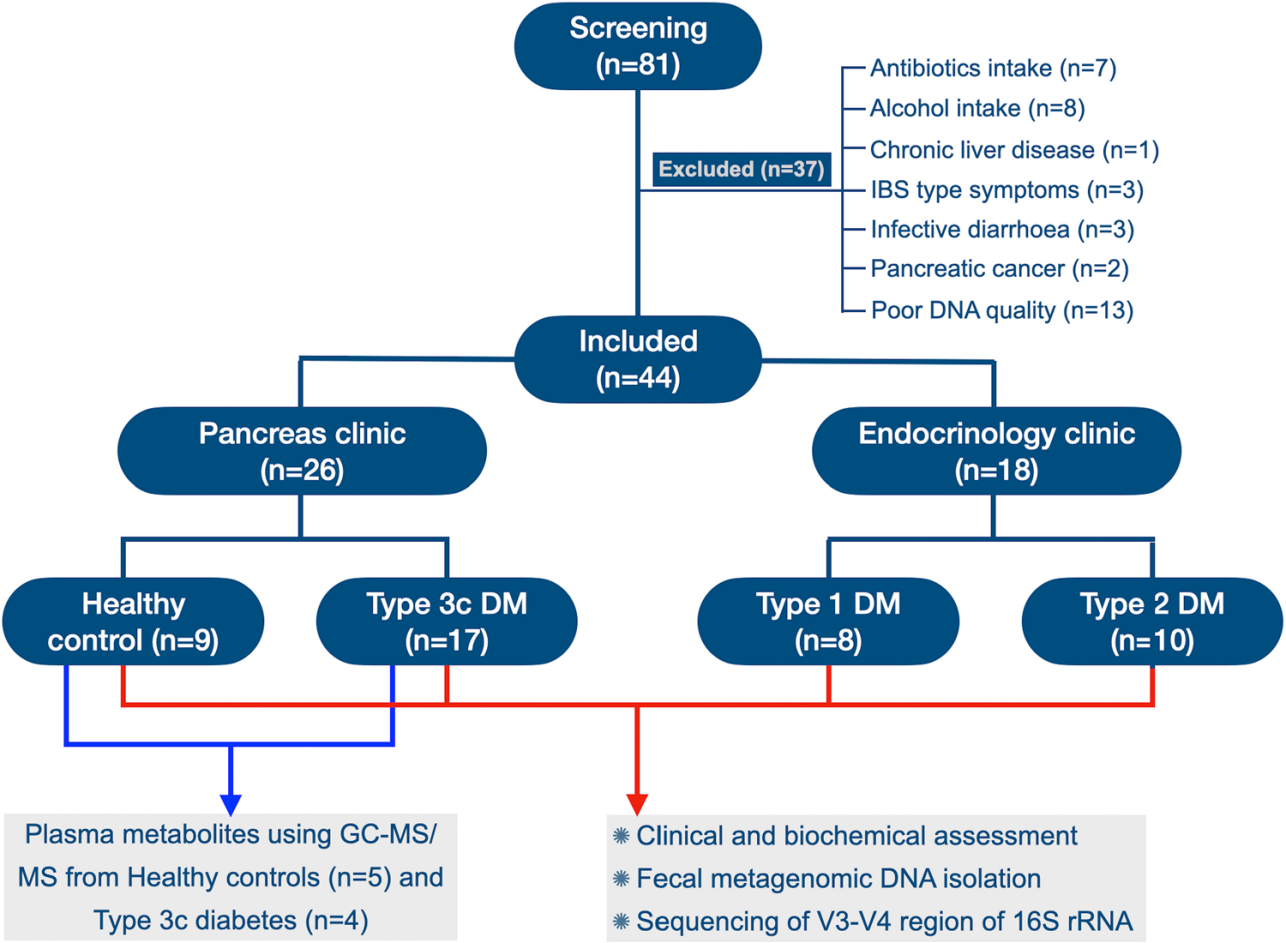
Study groups and patient distribution.

A total of 81 individuals were screened out of which 9 healthy volunteers, 8 patients with Type 1, 10 with Type 2 and 17 with Type 3c diabetes were finally included for analyses. The patient flow and clinical characteristics have been depicted in Fig. 1 and Supplementary Table 1 respectively. Supplementary Table 2 represents the metagenomic quality, number of sequences and singletons, and Good’s coverage of the fecal metagenome of individual control and patients.

### Fecal microbial diversity among study participants

**Figure 2:**
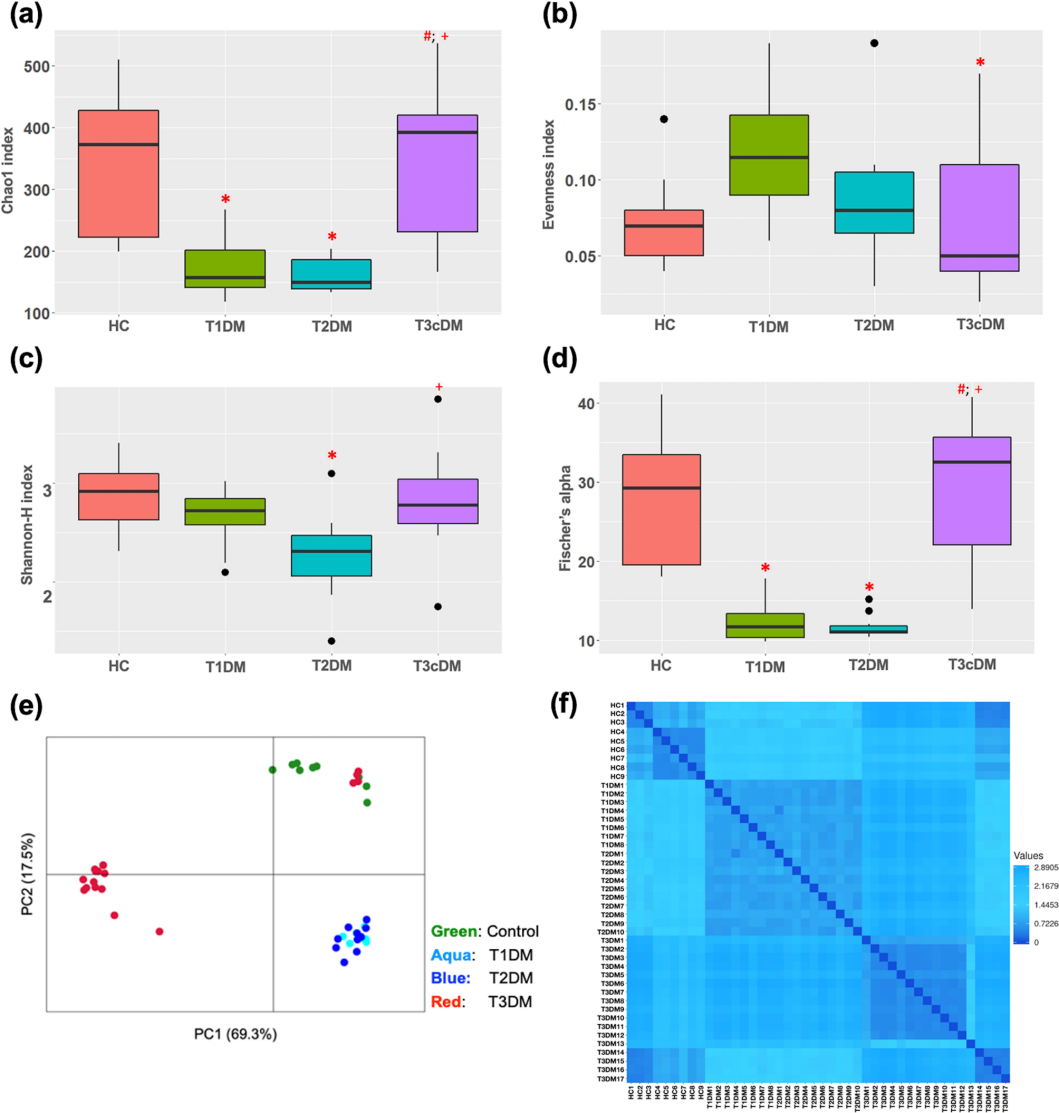
Fecal microbial diversity among the study patients. Box and whisker plots in panels a-d represent the alpha diversity indices. (**a**) Chao I, (**b**) Evenness index, (**c**) Shannon index, (**d**) Fischer’s alpha. Panels (**e**) Principal coordinate analyses [PCoA] plot using Euclidean distance and (**f**) Heatmap representing the intergroup and intragroup beta diversity, based on Euclidean distance, among the study groups. The *p* values were derived using the Kruskal–Wallis test with Tukey’s post-hoc pairwise analyses. * indicates statistically significant difference compared to healthy control; ^#^ compared to Type 1 diabetes and ^+^ compared to Type 2 diabetes. Refer to Supplementary Table 3 for exact *p* values.

The microbial alpha diversity indices and beta diversity are depicted in Fig. 2a–f and Supplementary Fig. 1. There were significant difference in the species richness (Chao 1) (*p* < 0.0001) as shown by a reduction among patients with Type 1 and Type 2 diabetes compared to healthy controls and those with Type 3c diabetes (adjusted *p* values for HC vs. T1DM = 0.03, HC vs. T2DM = 0.003, T1DM vs. T3cDM = 0.004 and T2DM vs. T3cDM < 0.0001, based on Tukey’s post hoc test) (Supplementary Table 3). Shannon index was found to be lowest in the patients with Type 2 diabetes compared to that of healthy controls and Type 3c diabetes (adjusted *p* values for HC vs. T2DM = 0.01, T2DM vs. T3cDM = 0.002, based on Tukey’s post hoc test) (Supplementary Table 3).

Figure 2e,f shows the beta diversity between the groups. The beta diversity was similar between the patients with Type 1 and Type 2 diabetes, while it was significantly different between patients with Type 3c diabetes and the other groups including healthy controls (Bonferroni corrected *p* values for HC vs. T1D = 0.001; HC vs. T2D = 0.001; HC vs. T3cD = 0.002; T1D vs. T3cD = 0.001, T2D vs. T3cD = 0.001).

### Differences in bacterial taxa between study groups

**Figure 3:**
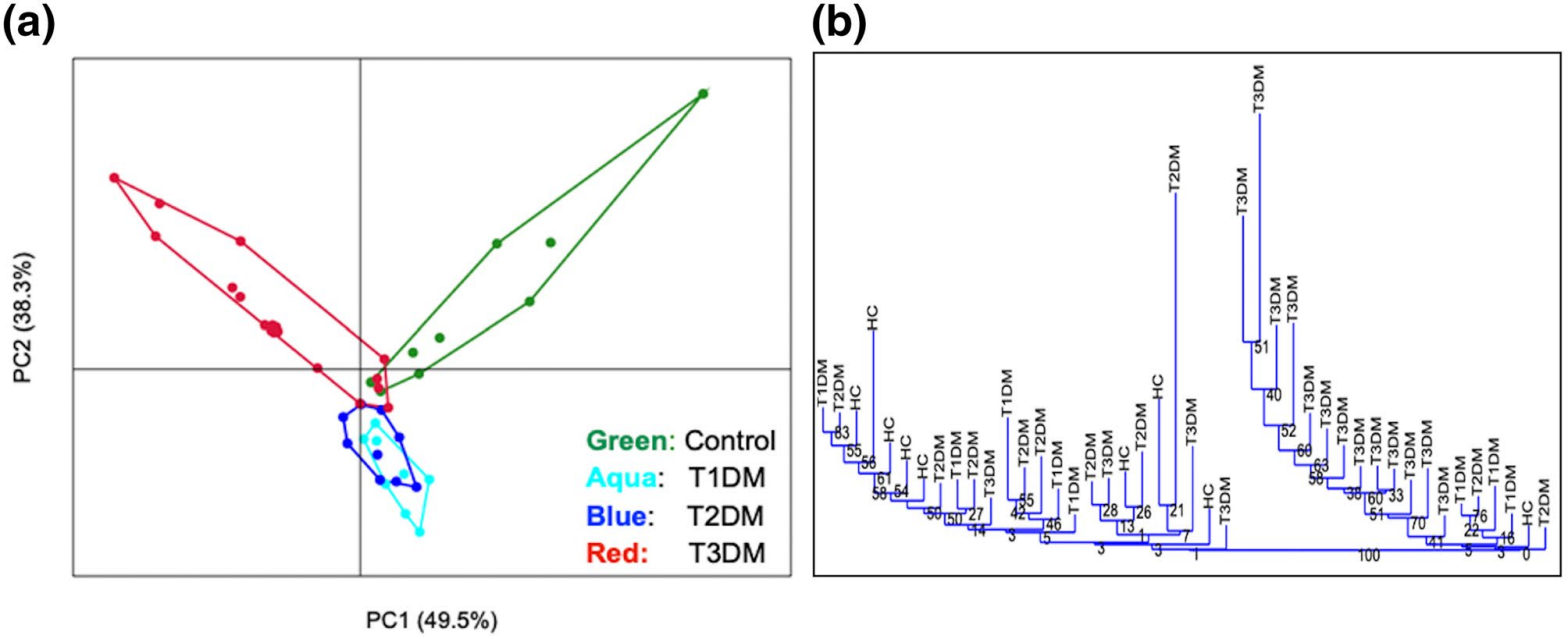
Clustering of gut microbiota at species level taxa among the study groups. (**a**) Principal component analysis (PCA) of gut microbial species abundance showing distinct clustering in the four study groups. The relation between the microbiota and disease status was assessed with PERMANOVA using 10,000 permutation [Bonferroni corrected *p* < 0.0001] followed by post hoc pair wise comparison. (**b**) Agglomerative clustering dendrogram showing hierarchical cluster analysis based on Euclidean similarity matrix (with boot strapping to 1000) to measure closeness between individual samples.

As shown in the PCA plot in Fig. 3a, there was significant differences in the species level taxa between Type 3c diabetes, healthy controls and, Type 1 and Type 2 diabetes combined (Bonferroni corrected *p* values for HC vs. T1D = 0.01; HC vs. T2D = 0.004; HC vs. T3cD = 0.002; T1D vs. T3cD = 0.009, T2D vs. T3cD = 0.002). This was confirmed in a hierarchical dendrogram (Fig. 3b). The scree plot for the PCA analyses have been depicted in Supplementary Fig. 2. The clustering of the different species between healthy controls and all diabetic patients, and between the three diabetic groups are shown in Supplementary Fig. 3.

**Figure 4:**
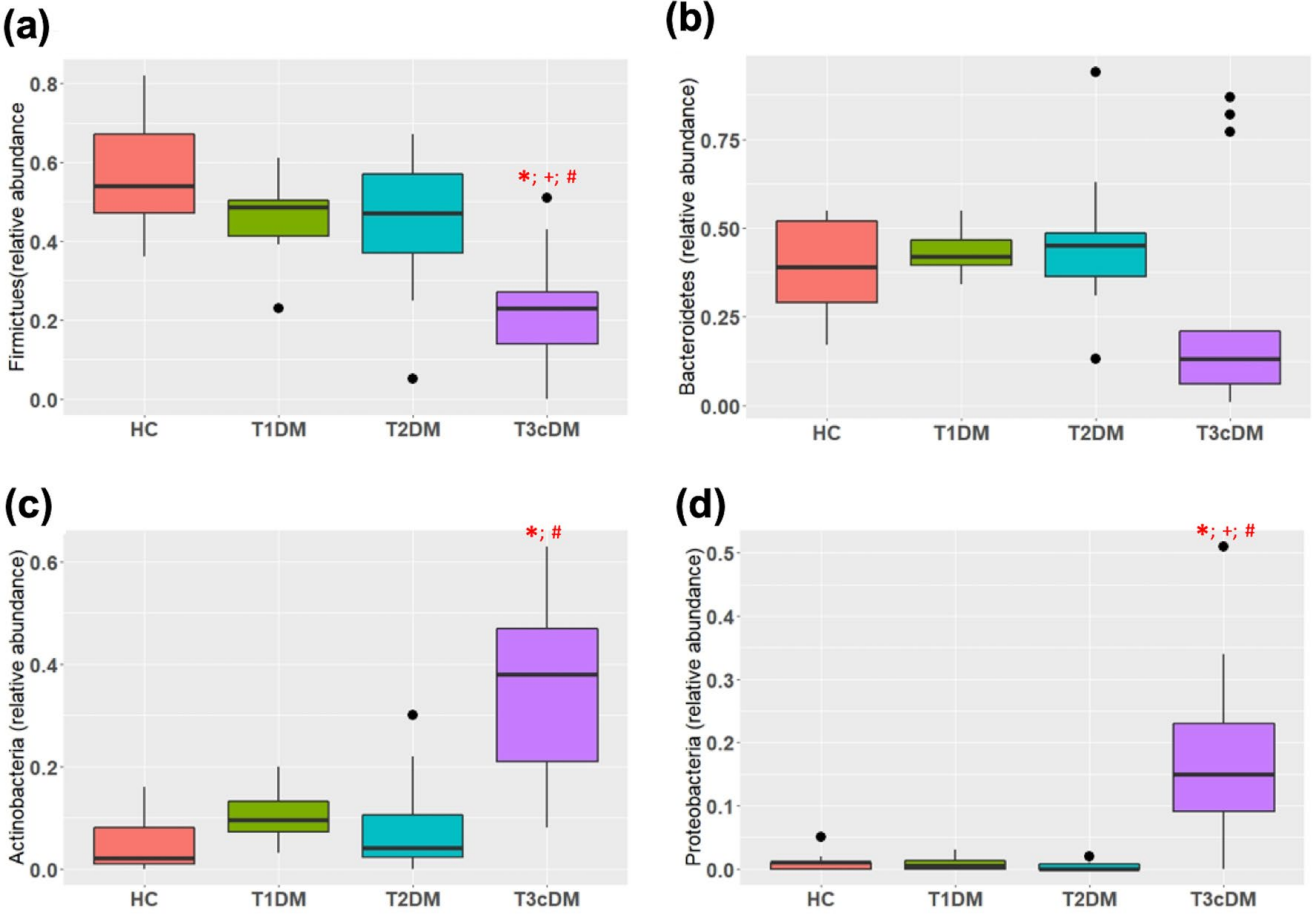
Relative abundances of the four most common phylum. Box and whisker plots depicting relative abundance of (**a**) Firmicutes; (**b**) Bacteroidetes; (**c**) Actinobacteria; (**d**) Proteobacteria. * indicates statistically significant difference between healthy control and T3c diabetes; ^+^ between Type 1 and Type 3c diabetes, ^#^ between Type 2 and Type 3c diabetes. Refer to Supplementary Table 4 for exact *p* values.

As shown in Fig. 4a–d, there were significant differences in the abundances in the phylum Firmicutes (*p* < 0.0001), Bacteroidetes (*p* = 0.04), Actinobacteria (*p* < 0.0001) and Proteobacteria (*p* < 0.0001), which together constituted the majority of the phyla. The Firmicutes to Bacteroidetes ratio was least in Type 3c diabetes compared to healthy controls (Supplementary Fig. 4). On post-hoc analyses (Supplementary Table 4), significant differences were observed in Firmicutes between Type 3c diabetes and healthy control (adjusted *p* < 0.0001), Type 1 diabetes (adjusted *p* = 0.03) and Type 2 diabetes (adjusted *p* = 0.01). Significant differences between these groups were also observed with Actinobacteria and Proteobacteria.

The relative abundances of organisms for each participant at the phylum, class, order and family level taxa has been depicted in Supplementary Fig. 5a–d respectively. Supplementary Fig. 6a–c shows the clustering of the study groups at the order, class and family level taxa while Supplementary Fig. 6d–e depicts abundance of these taxa according to the groups.

**Figure 5:**
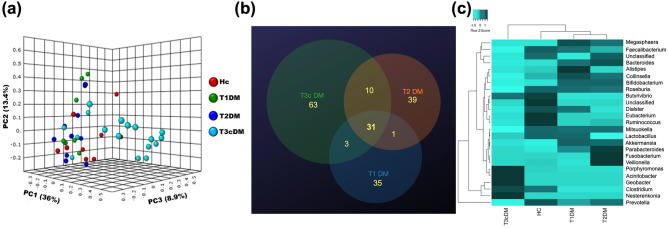
Bacterial characteristics at the genus level taxa. (**a**) PCoA plot, based on Bray Curtis distance matrix, showing significantly different clustering of Type 3c diabetes (Light blue dots) compared to the other groups (Red dots- healthy control, Green dots- Type 1 diabetes, Deep blue dots- Type 2 diabetes). (**b**) Venn diagram showing the numbers of unique and common genera between the three diabetes groups. (**c**) Heatmap with clustering dendrograms depicting the group wise difference in abundance of the organisms at the genus level taxa. The colour intensity representing the median relative abundances is based on the row z scores.

As shown in Fig. 5a, there was significant clustering of genus between the study groups with maximum difference for patients with Type 3c diabetes (*p* < 0.0001). The Venn diagram in Fig. 5b shows that there were 63 unique genera in the group of Type 3c diabetes while there were 35 and 39 in the Type 1 and Type 2 diabetes respectively. There were 31 genera common to all the three types of diabetes. Figure 5c and Supplementary Table 5 represents the abundances and intergroup differences of the top 25 genera. The core bacterial genera in the healthy control cohort included *Prevotella*, *Faecalibacterium*, *Roseburia, Eubacterium, Clostridium, Collinsella, Lactobacillus, Bacteroides, Ruminococcus, Parabacteroides, Dialister, Blautia, Butyricicoccus, Butyrivibrio, and Acetivibrio.* While, *Alistipes* was found uniquely in patients with Type 1 diabetes, both Type 1 and 2 group contained a core of *Prevotella*, *Faecalibacterium*, *Bacteroides*, *Collinsella*, *Ruminococcus*, *Clostridium*, *Eubacterium*, *Megasphaera, Bifidobacterium,* and unclassified Clostridiales. Patients with Type3c diabetes had a distinctive pattern of fecal bacteriome, containing *Nesterenkonia*, *Geobacter*, *Acinetobacter*, *Porphyromonas* and *Arthrobacter* as the unique core.

**Figure 6:**
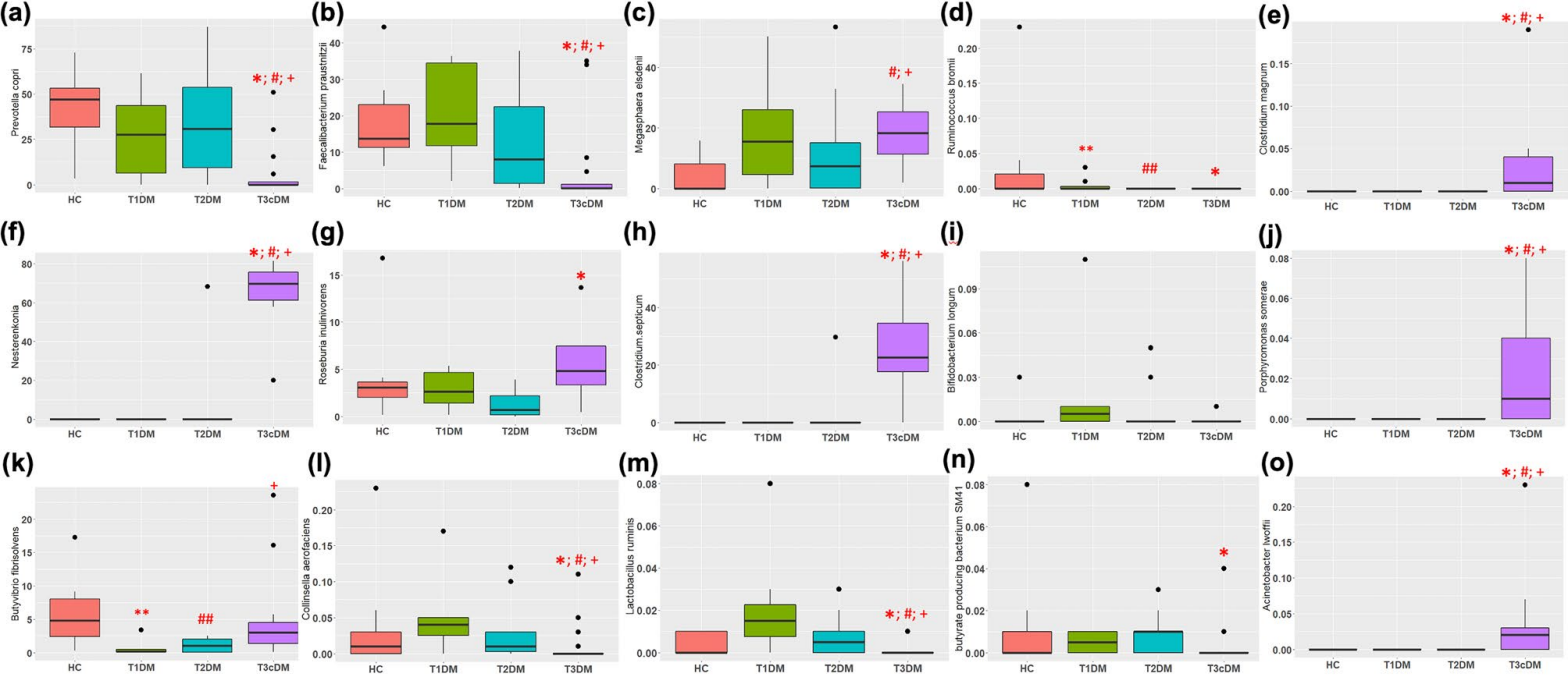
Relative abundances of top 15 species showing the most significant differences. Box and whisker plots depicting relative abundance of (**a**) *Prevotella copri*; (**b**) *Faecalibacterium praustnitzii*; (**c**) *Megasphaera elsdenii*; (**d**) *Ruminococus bromii*; (**e**) *Clostridium magnum*; (**f**) *Nesterenkonia.sp AN1*; (**g**) *Roseburia inulivorans*; (**h**) *Clostridium septicum*; (**i**) *Bifidobacterium longum*; (**j**) *Porphyromonas somerae*; (**k**) *Butyrivibrio fibrisolvens*; (**l**) *Collinsella aerofaciens*; (**m**) *Lactobacillus ruminis*; (**n**) *Butyrate producing bacteria SM41* (**o**) *Acinetobacter lwoffi*. * indicates statistically significant difference between healthy control and T3c diabetes; ** between healthy controls and Type 1 diabetes, ^#^ between Type 1 and Type 3c diabetes, ^##^ between healthy control and Type 2 diabetes, and ^+^ between Type 2 and Type 3c diabetes. Refer to Supplementary Table 6 for exact *p* values.

Supplementary Fig. 7 shows the top 25 species in the study groups while Fig. 6 and Supplementary Table 6 shows the abundances of the species with most significant difference. There was significant reduction of *Prevotella copri, Faecalibacterium praustnitzii, Collinsella aerofaciens* and *Lactobacillus ruminis* while significant increase uniquely in *Nesterenkonia *sp*. AN1, Clostridium magnum, Acinetobacter lwoffii, Clostridium septicum, Porphyromonas somerae, Terrabacter tumescens, and Synechococus *sp*.* in Type 3c diabetes compared to Type 1 and Type 2 diabetes. *Butyrivibrio fibrisolvens* was significantly low in abundance in Types 1 and 2 diabetes compared to healthy controls.

### Correlations between different genera in the study groups

**Figure 7:**
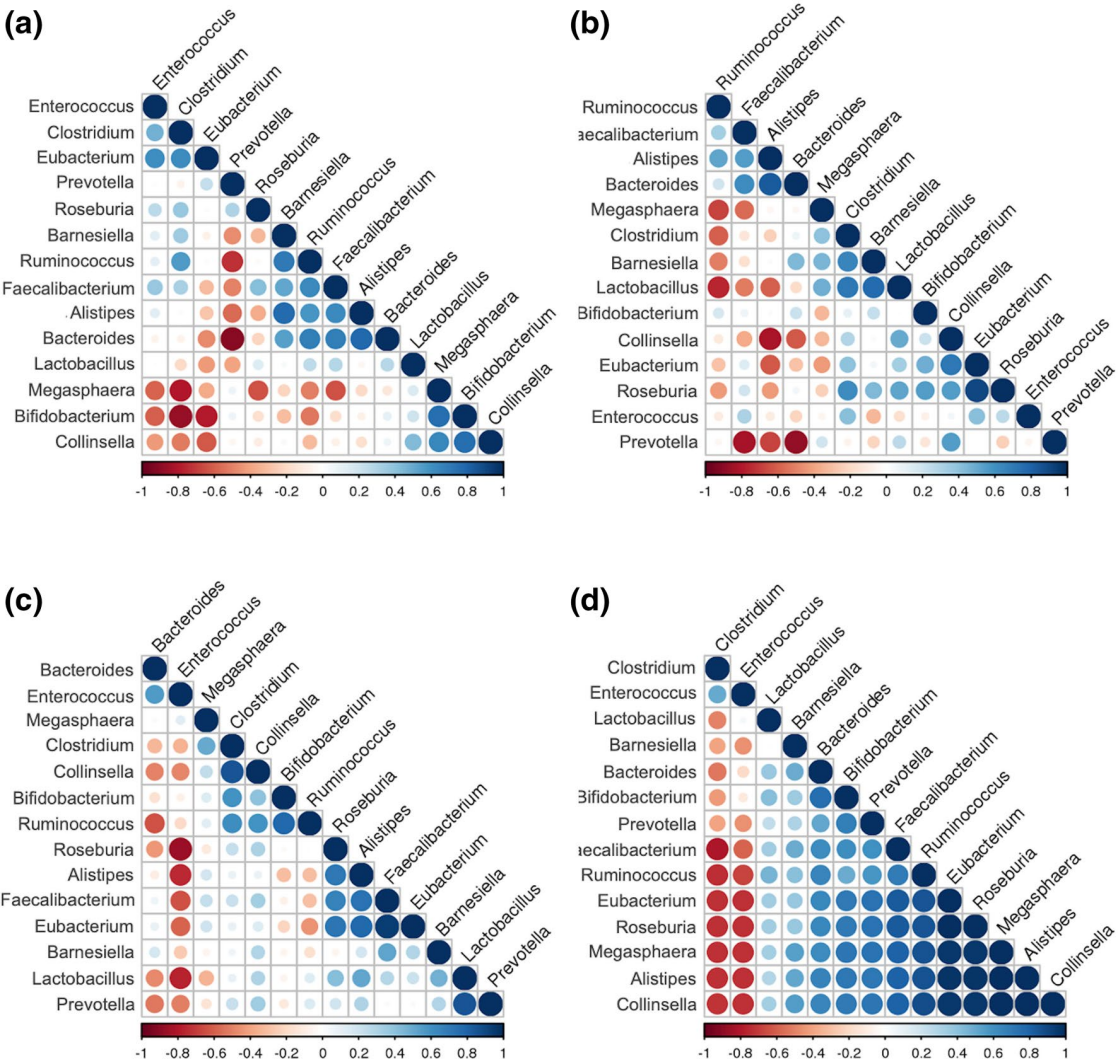
Corrogram depicting correlation of abundance of different genus. (**a**) Healthy control; (**b**) Type 1 diabetes; (**c**) Type 2 diabetes; (**d**) Type 3c diabetes.

As shown in Fig. 7, while there was a strong negative correlation between *Prevotella* and *Bacteroides* within the healthy control (*r* = − 0.90; *p* = 0.001) and Type 1 diabetes (*r* = − 0.88; *p* = 0.004) groups, the correlation was lost among patients with Types 2 and 3c diabetes. Furthermore, there was a strong negative correlation between *Bifidobacterium* and *Clostridium* among healthy controls (*r* = − 0.87; *p* = 002), which was not observed in the diabetes groups. In addition, among patients with Type 2 diabetes, there was a strong negative correlation of *Enterococcus* with *Lactobacillus* (*r* = − 0.77; *p* = 0.009) and *Roseburia* (*r* = − 0.86; *p* = 0.001). Furthermore, among patients with Type 3c diabetes, there were strong negative correlations of *Clostridium* with *Fecalibacterium* (*r* = − 0.80; *p* < 0.0001), *Ruminococcus* (*r* = − 0.74, *p* = 0.001), *Eubacterium* (*r* = − 0.73, *p* = 0.001), *Roseburia* (*r* = − 0.74; *p* = 0.001) and *Collinsella* (*r* = − 0.72; *p* = 0.001). There were also strong negative correlations of *Enterococcus* with *Eubacterium* (*r* = − 0.73, *p* = 0.001), *Roseburia* (*r* = − 0.73, *p* = 0.001) and *Collinsella* (*r* = − 0.73, *p* = 0.001) among the patients with Type 3c diabetes.

### Analysis of plasma metabolites

**Figure 8:**
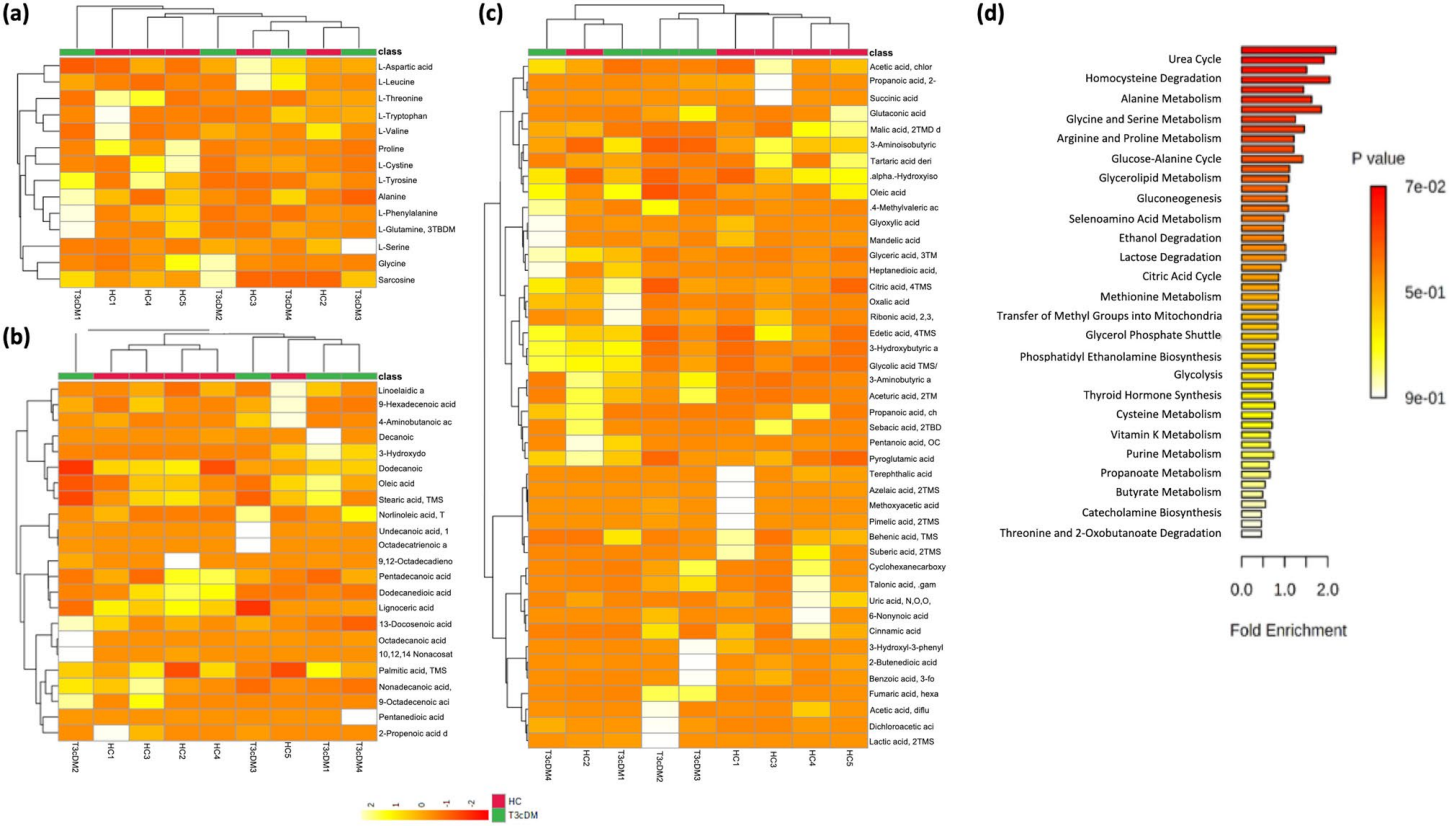
Metabolomic parameters of controls (n = 5) and patients with Type 3c diabetes (n = 4). Heatmaps with dendrograms depicting (**a**) Amino acid profile; (**b**) Fatty acid profile; (**c**) Non-fatty organic acid profile; Enrichment plot depicting (**d**) Metabolic pathways.

We conducted preliminary evaluation for plasma metabolites and pathway enrichment analyses (Fig. 8a–d; Supplementary Fig. 8) in 5 healthy controls and 4 patients with Type 3c diabetes. Although we did not find any significant unique metabolite signature based on the type of diabetes, we did observe a trend towards reduction of l-threonine, l-cystine, l-phenylalanine in Type 3c diabetes compared to healthy controls. Moreover, patients with Type 3c diabetes showed an increasing trend for fatty acids and sphingolipids (eg. myoinositol) compared to the healthy controls. As shown in Supplementary Fig. 9, we could not demonstrate statistically significant differences in the interactions between the microbiome and the metabolome.

## Discussion

In this preliminary cross-sectional study, we have shown that patients with Type 3c diabetes has a unique gut bacterial signature that differs from Type 1 and Type 2 diabetes. Since the gut microbiota is shaped by a variety of physiological and pathological factors, and the pathophysiology of the three types of diabetes are different, we hypothesized that gut microbial dysbiosis in these patients could also be variable. This speculation was triggered by the results from our earlier studies on CP which suggested that Type 3c diabetes was associated with islet inflammation and infiltration with Th17 cells^26–28^. It was also suggested that islet inflammation could be contributed by endotoxemia that resulted from gut microbial dysbiosis and altered gut mucosal permeability in these patients^20^. Even though there are earlier studies that had evaluated the gut microbiome in diabetes, to the best of our knowledge this the first study to have compared the gut microbiome head to head in the three types of diabetes. The other factor that makes the current study relevant is the recent evidence that confirms the Indian gut microbiota to be unique from that seen in the west^29^. Dubey et al. reported 993 unique organisms in a pan Indian study of which 390 were common to all geographic locations and the most abundant species were *Prevotella copri* and *Faecalibacterium praustnitzii*^30^.

In the current study, the predominant organisms in the healthy controls included *Prevotella, Fecalibacterium, Ruminococcus, Roseburia,* which were in alignment with the earlier reports of normal Indian gut microbiota^29,31,32^. In the Type 2 diabetic group, we observed a reduction in abundance in *Fecalibacterium, Eubacterium, and Ruminococcus* compared to controls, which was similar to that observed in earlier studies on Indian Type 2 diabetes patients^17,18^. Our current results were also similar to our earlier report in which we demonstrated significant reduction in *Fecalibacterium praustnitzii* and *Ruminococcus bromii* in patients with CP with diabetes^20^. These data had ensured the reliability of the findings on altered microbiome in the diabetic patients in this study. There are so far no Indian studies that reported on gut microbiome in Type 1 diabetes. In the current study, we observed reduced abundance of *Prevotella* and *Faecalibacterium* while increase in *Bacteroides, Alistepes*, *Bifidobacterium* and *Parabacteroides* that was similar to studies from the west^13,21^.

Although we observed significant difference in the dysbiosis between the three type of diabetes, it would be difficult to comment on the cause-effect relationship sans experimental evidence. Nevertheless, the dysbiosis that we observed is likely to have functional implications in the pathophysiology of diabetes. An increased abundance of *Bacteroides* sp. have been shown to result in altered mucosal integrity and gut permeability by affecting zonulin level, that further modulates the intercellular junctions and macromolecular passages in the cell^15^. Additionally, a higher abundance of *Vellionella* sp. could impact host health by producing lactate which eventually could weakens cellular tight junctions. Further, the reduction of butyrate producing genera *Faecalibacterium*, *Ruminococcus*, and *Eubacterium* is known to result in a proinflammatory environment within the gut as butyrate induces colonic T-reg cells, decreases pro-inflammatory macrophage production, thereby enhancing the gut barrier integrity. It also enhances mucin production which is also helpful in maintaining the gut permeability^33^. Additionally *R*. *bromii* has been considered as a keystone species which metabolizes starch and the reduction of the species may lead to the poor starch degrading capacity of the host, which is often observed in the Type 3c diabetics^34^. All these genera were significantly altered in the patients with Type 3c diabetes compared Type 1 and Type 2 diabetes.

Recent studies have shown that *Prevotella copri* is among the most abundant core organism in the heathy Indian gut^29–31^. We observed a significant reduction in the abundance of this organism in patients with Type 3c diabetes compared to healthy control, Type 1 and Type 2 diabetes. While we did not observe any difference of *Prevotella copri* between healthy controls and Type 1 diabetics, it was earlier reported to be increased in Type 1 diabetics^15^. *Prevotella* has been linked to produce propionate, succinate and acetate which are responsible for gut mucosal and tight junction integrity, and T-reg differentiation. *Prevotella* has been linked with a diet rich in plant derived carbohydrates and fiber and is in inverse relationship with *Bacteroides*^35^. The high abundance of *Prevotella* in our healthy controls can be explained by a vegetarian predominant diet in India, and the loss of correlation of Prevotella and Bacteroides in the Type 3c diabetes group (Fig. 3) suggests its potential implication in Type 3c diabetes.

We observed significantly lower abundance of *Megasphaera* in Type 3c diabetes compared to the patients with Type 1 and Type 2 diabetes. It has now been shown that *Megasphaera elsdenii, Megasphaera *sp*. NM10* and *Megasphaera *sp*. BL7* renders a positive effect to the host health by utilizing lactate to produce SCFAs and synthesizing riboflavin^36^. This points towards a potential implication of reduced *Megasphaera* in Type 3c diabetes. We also observed unique bacterial profiles in Type 3c diabetes group namely, *Nesterenkonia* sp., *Geobacter* sp., *Clostridium* sp. *Acinetobacter* sp., and *Porphyromonas* sp. *Porphyromonas somarae* has been linked with an increase in pro-inflammatory cytokines. This may have an implication in Type 3c diabetes since it has been associated with chronic inflammation of the pancreas. *Nesterenkonia* sp. has also been considered as pathogenic and has been shown to be associated with the inflammatory condition in the host^37^.

CP is characterized by chronic, progressive inflammation involving the exocrine and endocrine pancreas. It is characterized by exocrine insufficiency that results in fat malabsorption. Excess fat in the intestine results in dysbiosis, which could eventually lead to intestinal inflammation, gut barrier dysfunction, and endotoxemia^38^. Circulating endotoxin could eventually cause islet inflammation via TLR4 involving the NFkB pathway^39^. The resulting hyperglycemia could further lead to gut microbial dysbiosis, resulting in a vicious cycle. This hypothesis appears to explain the significant differences in the gut microbiome in patients with Type 3c diabetes compared to Type 1 and 2 diabetes Furthermore, patients with CP are associated with intestinal dysmotility and small intestinal bacterial overgrowth, irrespective of glycemic status, and the morphological and biochemical characteristics of CP are heterogenous^40,41^. This could further explain our observation that the dysbiosis was particularly high in some of the Type 3c diabetics compared to others within the group, and the higher abundance of the phylum Proteobacteria was particularly high in this group. Further support to our observation comes from the fact that these patients had a poorer glycemic control. However, this hypothesis needs to be confirmed by experimental approaches using established models of CP.

A drawback of our study is the small sample size. Since there were no prior comparative studies, we decided to initially conduct a preliminary study. Since the results are encouraging, a better structured study with a large sample size is now mandated. Moreover, we did not observe significant differences in the metabolome profile in spite of differences in the bacteriome profile. This could also be explained by the very small sample size. Since there were some trend in significance in a few of the metabolites, it is likely that studies with larger sample sizes would yield meaningful differences. Furthermore, use of other techniques such as LC–MS/MS could also provide plausible results.

In conclusion, in this preliminary cross-sectional study, we report significant differences in gut microbiome in patients with Type 3c or pancreatogenic diabetes compared to patients with Type 1 and 2 diabetes. Our data needs to be validated in larger multicentre cohorts of patients and the cause-effect relationship needs to be evaluated in experimental studies in order to make the results generalizable.

## Methods

### Patient recruitment and clinical data acquisition

This preliminary cross-sectional study was conducted at a tertiary care academic centre in accordance with principles of the Declaration of Helsinki as revised in 2008. Ethical approval was obtained from the AIG Institutional Review Board prior to initiation of the study, and written informed consent was taken from all the participants prior to recruitment. All experimental procedures were performed according to standard guidelines and procedures that were approved by the Asian Institute of Gastroenterology Institutional Review Board. Patients with CP of at least 3 yrs duration and having poorly controlled Type 3c diabetes were screened for enrolment criteria in the Pancreas Clinic over a 9-month period. Patients with poorly controlled Type 1 and Type 2 diabetes were recruited from the Endocrinology outpatient clinic over a 3-month period. Individuals with history of inflammatory bowel disease, irritable bowel syndrome, chronic liver disease, recent critical illness, ongoing pregnancy, constipation and diarrhoea in the past 3 months, and antibiotics and probiotics intake in the past 3 months were excluded. We recruited healthy family members between 18 and 60yrs age who lived with the patients for at least 10 years as controls. Patients and healthy controls who were obese, smoked cigarettes, consumed alcohol and had poor quantity/quality metagenomic DNA were also excluded.

Data pertaining to demographic characteristics of patients and controls, diabetic status, clinical and imaging parameters of the patients with CP, details of treatment of diabetes and CP were captured in a structured proforma.

### Definitions

CP was defined as per the MANNHEIM classification and confirmed with contrast enhanced computed tomography (CECT) scan^42^. Severity of CP was defined using magnetic resonance cholangiopancreatography (MRCP) using the Cambridge criteria for endoscopic retrograde cholangiopancreatography (ERCP) or endoscopic ultrasonography (EUS) using the Rosemont criteria^43^. The different types of diabetes were defined according to the ADA 2014 criteria^44^. We adopted the term Type 3c diabetes for diabetes secondary to exocrine pancreas as per recent literature. We considered the diagnosis of Type 3c diabetes in the patients with CP if it was diagnosed after a consistent lag period following the onset of CP and had a low to low-normal plasma C-peptide levels. Poorly controlled diabetes was defined as HbA1c of 7% and/or preprandial capillary plasma glucose between 80 and 130 mg/dL and/or peak postprandial capillary plasma glucose of 180 mg/dL.

### Sample collection, biochemical evaluation, imaging and treatment

After recording clinical data, fecal samples were collected in a sterile fecal container, containing 3 ml RNA Later (Qiagen, Germany cat.no/ID: 76,106). Blood samples were collected after 8 h of fasting and 2 h post meal in K3-EDTA tubes for estimation of blood glucose, glycosylated hemoglobin (HbA1c) and C-peptide. Blood samples were also collected for evaluation of plasma metabolites in a proportion of patients and healthy controls. Both fecal and plasma samples were stored at − 80 °C immediately after collection until further analysis.

We estimated blood glucose using the Glucose Oxidase–Peroxidase (GOD-POD) method with ERBA Glucose kit (Transasia Bio-Medicals Ltd, HP, India). High performance liquid chromatography (HPLC) using a National Glycohemoglobin Standardised Program (NGSP) certified automated analyser from Bio-Rad was used to measure HbA1c. Plasma C-peptide assay was estimated using the sandwiched Electrochemical Immunoassay (ECLIA) technique in the Roche Cobas ‘e’ 601 Immunoassay Analyzer. Briefly, double incubations were done with 20 μl biotinylated monoclonal antibodies (anti C-peptide) followed by streptavidin coated micro particles. The bound micro particles were then magnetically captured onto the surface of the electrode that resulted in induction of chemiluminescent emission upon current application. The emission was measured on a photomultiplier and quantified by 2-point calibration with a master curve.

Disease morphology in the patients with CP was evaluated by cross sectional imaging [contrast enhanced computed tomography (CECT) or magnetic resonance cholangiopancreatography (MRCP)] and endoscopic retrograde cholangiography (ERCP). All controls and patients with Type 1 and Type 2 diabetes underwent transabdominal ultrasonography to evaluate for the pancreas and the liver.

Patients with CP were treated with antioxidant cocktails, pancreatic enzyme supplementation and analgesics on demand. Patients who had recurrent intractable pain that did not respond to medical therapy were treated with endoscopic treatment. Patient with Type 1 diabetes were treated with insulin, while patients with Type 2 and Type 3c diabetes were initiated on oral hypoglycemic agents (OHAs). If glycemic control was suboptimal with OHAs, then insulin treatment was initiated.

### Fecal DNA extraction

The metagenomic DNA isolation was performed (200 mg stool/sample) using Qiagen mini stool DNA isolation kit (Germany, Cat. No 81504) as per the manufacturer’s instruction. DNA quantity and quality were assessed using the Nano drop 2000 spectrophotometer (Thermo Scientific, IL, USA) based on A260/A280 absorbance and agarose gel electrophoresis (1% wt vol^−1^ agarose in Tris acetate EDTA). Reagents from the same lot was used for all pre-sequencing sample processing.

### Next generation sequencing (NGS)

NGS of the V3–V4 region of 16SrDNA in the fecal metagenome was conducted on the Illumina MiSeq platform at Xcelris Genomics (Ahmedabad, India) with 2*250 (i.e. paired end) chemistry. The paired end sequence data were assembled into contigs following V3–V4 region amplification using the universal primers (V3-F: 5′ CCTACGGGNGGCWGCAG3′ and V4-R: 5′GACTACHVGGGTATCTAATCC3′). The subsequent steps such as ligation with the adaptors, library preparation, barcode additions were processed as described previously^20^. The R1 and R2 reads from each sample were stitched or assembled and then proceeded for quality check i.e. phred (Q20) quality score, and sequences with less than Q20 were removed. Chimeric sequences, mismatched sequences, barcodes and the sequences with length less than 100 bp were also removed from the final sequences, following which the rest of the reads were uploaded to the MG-RAST server^45^. The metadata and raw sequences have also been submitted to the NCBI SRA portal with BioProject accession PRJNA723868 (SRR1430686-SRR1430729). Taxonomic assignment was performed with 95% similarity cut off with the RDP (Ribosomal Database Project) database in the MG-RAST server. We used positive and negative controls in the sequence runs to evaluate for batch effect and inadvertent contamination respectively.

### Sample preparation for metabolite analysis

Plasma was separated within 30 min of blood withdrawal from the study participants. Plasma metabolite profiles were screened using the Gas Chromatography Mass Spectrometry (GCMS). Briefly, 300 µl of plasma samples were extracted in n-methanol (1:1, vv^−1^) followed by 15 h incubation on a shaker incubator at room temperature. Samples were centrifuged at 12,000 rpm for 5 min at 4 °C. A 200 µl extract was preserved for drying in a desiccator for 24 h. Derivatization was performed using 50 µl of Pyridine (20 mg/ml, Sigma-Aldrich, (CAS nos. 110-86-1) followed by incubation at 30 °C for 90 min and 30 µl N-trimethylsilyl-N-methyl trifluoroacetamide (MSTFA, Sigma-Aldrich, CAS no. 24589-78-4) followed by re-incubation at 37 °C for 30 min. We did not use any internal standard since the study was focused on untargeted metabolite profiling of the samples.

### Gas Chromatography mass spectrometry (GCMS)

1 µl of the derivatized samples were prepared for GCMS analysis under the Splitless injection condition as per the program described earlier. We used a triple quadrupole Shimadzu GC 2010 Plus-TP-8030 system (equipped with EB5MS column), and helium was used as the carrier gas. The peak area for each metabolite which was based on the m/z ratio and peak intensity was considered for further analysis. Background peaks and column bleed peaks consisting of silanes and siloxanes were not considered. The peak area of a single compound showing multiple derivatized forms were pooled for the downstream analysis. National Institute of Standards and Technology (NIST) library was used to identify the peaks. Further functional analysis of the metabolite was carried out using the human metabolite database (HMDB) (http://www.hmdb.ca)^46^ and the Kyoto Encyclopedia of Genes and Genomes database (KEGG, http://www.genome.jp/kegg/)^47^.

### Statistical analyses

Since there are no prior parallel comparison of the gut microbiota between the three types of diabetes, we decided to undertake the current comparison as a preliminary study. Therefore, we did not perform any formal sample size calculation. A database was generated in Excel for Mac and statistical analyses were conducted using the Statistical Package of Social Scientists (SPSS) (IBM SPSS 20, SPSS Inc, Chicago, IL), Paleontological Statistics Software (PAST) (Version 3.11 for Mac)^48^ and R studio (Version 1.3.959)^49^ statistical platforms. Continuous clinical data were expressed as mean (± SD) while categorical data were expressed as proportions.

Microbial richness and alpha diversity were expressed as Chao 1, and Shannon-H index, Evenness index, Fischer’s alpha respectively based on the species level abundance. The data were represented as box and whisker plots generated in ggplot2 package for R. Beta diversity was assessed by principal coordinate analysis calculating the Euclidean distance matrix. Per sample rarefaction curves were constructed using the MicrobiomeAnalyst online platform based on minimum library depth after normalization to rule out artifacts, as per the method described by Weiss et al.^50^. The initial comparison among the microflora in all the groups were made using the absolute abundance /counts of the taxa in each group. Principal Component analysis (PCA) with convex hulls was created to evaluate the intergroup differences and the groups were compared by one-way permutational multivariate analysis of variance (PERMANOVA) using 10,000 permutations with species abundance values in PAST (PAleontological STatistics software, Mac version 3.11). Significant differences in the microbiome profiles were tested using the non-parametric Kruskal–Wallis test (KW) followed by Tukey’s post-hoc pairwise analyses in SPSS with Bonferroni corrections for multiple hypothesis testing. Relative abundances of the different taxonomic levels, (i.e. phylum, class, order, family, genus and species) were expressed by box and whisker plots. Heatmaps were constructed based on the abundance value for microbes and area value of the peaks detected by GCMS for metabolites in R script based Metaboanalyst 4.0 and Heatmapper data expression tools^51,52^. The correlation matrices were generated using Spearman correlation in SPSS and the matrices were visualized as corrograms with the R script based STHDA online platform^53^. Venn diagram was used to express common genera among the different groups using the Venn diagram maker^54^. Microbiome-metabolome correlation networks were generated using Cytoscape v 3.8^55^. The pathway enrichment plots were generated using the MetaboAnalyst v.4.0.

## Supplementary information

Supplementary Information.

## Abbreviations

CECT: Contrast enhanced computed tomography
CP: Chronic pancreatitis
ECLIA: Electrochemical immunoassay
ERCP: Endoscopic retrograde cholangiopancreatography
EUS: Endoscopic ultrasound
GOD-POD: Glucose oxidase–peroxidase
KEGG: Kyoto encyclopedia of genes and genomes database
MG-RAST: Metagenomic rapid annotations using subsystems technology
MRCP: Magnetic resonance cholangiopancreatography
NGS: Next generation sequencing
NGSP: National glycohemoglobin standardised program
OHA: Oral hypoglycemic agents
PAST: Paleontological statistics software
PCA: Principal component analysis
PERMANOVA: Permutational multivariate analysis of variance
SPSS: Statistical package for social scientists
Th: T-helper
