# Supplementary material for: The gut microbiome in pancreatogenic diabetes differs from that of Type 1 and Type 2 diabetes
Source: Sci Rep. 2021 May 26;11:10978. doi: 10.1038/s41598-021-90024-w (PMC8155207; doi:10.1038/s41598-021-90024-w)

**The gut microbiome in pancreatogenic diabetes differs from that of Type 1 and Type 2 diabetes: a preliminary study.**

Rupjyoti Talukdar^1,2^, Priyanka Sarkar^1^, Aparna Jakkampudi^1^, Subhaleena Sarkar^1^, Mohsin Aslam^3^, Manasa Jandhyala^1^, Deepika G^4^, Misbah Unnisa^2^, D. Nageshwar Reddy^2^.

^1^ Wellcome DBT India Alliance Labs.

Institute of Basic and Translational Research, Asian Healthcare Foundation

Asian Institute of Gastroenterology

Hyderabad

^2^ Dept. of Medical Gastroenterology

Asian Institute of Gastroenterology

Hyderabad

^3^ Dept. of Endocrinology,

Asian Institute of Gastroenterology

Hyderabad

^4^ Dept. of Biochemistry,

Asian Institute of Gastroenterology

Hyderabad

**Supplementary Table 1:** Clinical characteristics of the patients and controls at baseline.

|  | **Healthy control (n=9)** | **Type 1 DM (n=8)** | **Type 2 DM (n=10)** | **Type 3c DM (n=17)** |
| --- | --- | --- | --- | --- |
| **Age in years (mean; SD)** | 42.9 (13.1) | 20.9 (4.5) | 49.2 (7.2) | 35.1 (11.9) |
| **Male gender (n; %)** | 6 (66.7) | 5 (63.5) | 7 (70) | 13 (76.5) |
| **BMI (kg/m^2^) (mean; SD)** | 21.3 (1.7) | 19.2 (2.3) | 21.6 (1.7) | 20.3 (1.8) |
| **Non-vegetarian diet (n; %)** | 9 (100) | 4 (50) | 6 (60) | 11 (64.7) |
| **Duration of CP in years (mean; SD)** | NA | NA | NA | 6.6 (2.7) |
| **Idiopathic etiology (n; %)** | NA | NA | NA | 14 (82.3) |
| **Gross pancreatic atrophy (n; %)** | NA | NA | NA | 10 (58.8) |
| **Pancreatic calculi/calcification (n; %)** | NA | NA | NA | 11 (64.7) |
| **Duration of diabetes in years (mean; SD)** | NA | 8.2 (3.6) | 7.0 (4.4) | 1.4 (1.4) |
| **Fasting blood glucose (mg/dl) (mean; SD)** | 92.2 (4.3) | 137.9 (22.7) | 133.4 (8.9) | 144.0 (26.3) |
| **2hrs PP blood glucose (mg/dl) (mean; SD)** | 128.0 (14.9) | 190.8 (36.0) | 184.8 (37.9) | 210.3 (38.2) |
| **HbA1c (Mean; SD)** | 5.2 (1.1) | 7.5 (0.4) | 7.8 (0.6) | 8.2 (0.9) |
| **Insulin requirement (n; %)** | NA | 8 (100) | 4 (40) | 6 (35.3) |

**Supplementary Table 2:** Sequence characteristics, metagenomic quality, richness and alpha diversity of intestinal microbiota in controls and patients with diabetes.

| **Group** | **SRA accession no**. | **DNA quality (A260:280)** | **Total no. of sequences** | **No. of QC failed sequences** | **No. of singleton** | **Good's coverage** |
| --- | --- | --- | --- | --- | --- | --- |
| **Healthy control** | SRR14307729 | 1.99 | 299523 | 0 | 23 | 99.99 |
|  | SRR14307728 | 2.13 | 227429 | 0 | 29 | 99.99 |
|  | SRR14307717 | 2.06 | 223147 | 0 | 31 | 99.98 |
|  | SRR14307706 | 2.17 | 346181 | 172 | 78 | 99.98 |
|  | SRR14307695 | 2.07 | 515969 | 787 | 64 | 99.99 |
|  | SRR14307690 | 2.12 | 331296 | 597 | 52 | 99.98 |
|  | SRR14307689 | 2.14 | 893828 | 2,659 | 77 | 99.99 |
|  | SRR14307688 | 2.13 | 388653 | 888 | 59 | 99.99 |
|  | SRR14307687 | 2.15 | 339207 | 1,456 | 63 | 99.98 |
| **Type1 DM** | SRR14307686 | 2.03 | 275400 | 733 | 38 | 99.98 |
|  | SRR14307727 | 2.09 | 474259 | 1,593 | 23 | 99.99 |
|  | SRR14307726 | 2.02 | 382807 | 728 | 26 | 99.99 |
|  | SRR14307725 | 2.01 | 440732 | 413 | 20 | 99.99 |
|  | SRR14307724 | 2.1 | 178313 | 421 | 14 | 99.99 |
|  | SRR14307723 | 2.02 | 295678 | 326 | 45 | 99.99 |
|  | SRR14307722 | 1.99 | 421204 | 378 | 24 | 100.00 |
|  | SRR14307721 | 2.15 | 237175 | 414 | 31 | 99.99 |
| **Type2 DM** | SRR14307720 | 1.88 | 494381 | 906 | 17 | 99.99 |
|  | SRR14307719 | 1.91 | 498329 | 1,190 | 29 | 99.99 |
|  | SRR14307718 | 2.08 | 336391 | 292 | 37 | 99.99 |
|  | SRR14307716 | 1.84 | 378785 | 576 | 31 | 99.99 |
|  | SRR14307715 | 2.04 | 631419 | 1,337 | 32 | 100.00 |
|  | SRR14307714 | 1.84 | 345954 | 483 | 21 | 99.99 |
|  | SRR14307713 | 1.99 | 376992 | 992 | 28 | 99.99 |
|  | SRR14307712 | 2.03 | 400284 | 985 | 24 | 99.99 |
|  | SRR14307711 | 2.06 | 198952 | 409 | 24 | 99.98 |
|  | SRR14307710 | 2.18 | 340491 | 228 | 31 | 99.99 |
| **Type3c DM** | SRR14307709 | 2.15 | 17823 | 205 | 46 | 99.78 |
|  | SRR14307701 | 1.88 | 227633 | 17 | 67 | 99.97 |
|  | SRR14307700 | 2.07 | 265863 | 16 | 66 | 99.98 |
|  | SRR14307699 | 2.16 | 312214 | 10 | 67 | 99.98 |
|  | SRR14307698 | 1.86 | 232971 | 11 | 75 | 99.97 |
|  | SRR14307697 | 2.04 | 187474 | 8 | 64 | 99.97 |
|  | SRR14307708 | 2.02 | 351524 | 478 | 50 | 99.99 |
|  | SRR14307707 | 2.22 | 548425 | 645 | 62 | 99.99 |
|  | SRR14307705 | 2.08 | 319541 | 208 | 93 | 99.98 |
|  | SRR14307704 | 2.12 | 671918 | 764 | 73 | 99.99 |
|  | SRR14307703 | 2.07 | 401376 | 369 | 72 | 99.98 |
|  | SRR14307702 | 2.16 | 849150 | 913 | 79 | 99.99 |
|  | SRR14307691 | 2.08 | 440691 | 654 | 22 | 99.99 |
|  | SRR14307696 | 2.11 | 547872 | 3 | 25 | 100.00 |
|  | SRR14307694 | 2.12 | 224187 | 0 | 19 | 99.99 |
|  | SRR14307693 | 2.16 | 254653 | 1 | 29 | 99.99 |
|  | SRR14307692 | 2.05 | 307061 | 1 | 19 | 99.99 |

**Supplementary Figure 1:** Diversity indices for each individual in the study groups. **(a)** Rarefaction curves; **(b)** Chao I; **(c)** Evenness index; **(d)** Shannon-H index; **(e)** Fischer’s alpha.

**
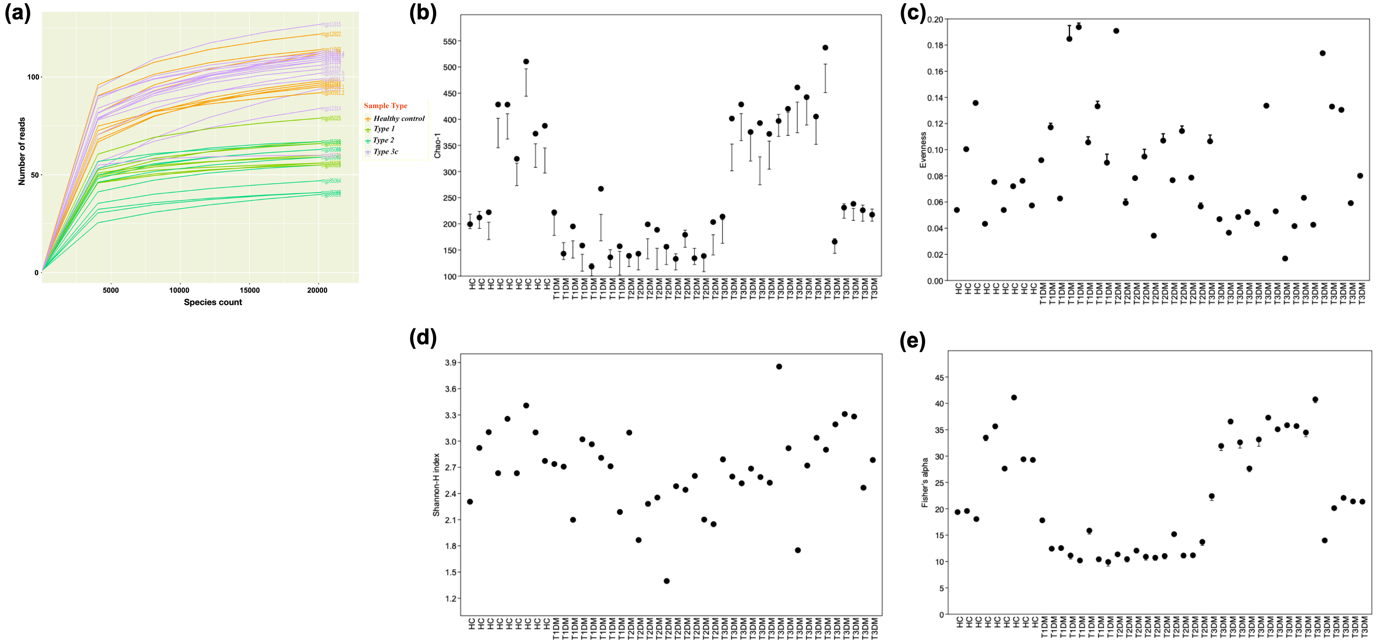
**

**Supplementary Table 3**: Significance levels of differences in alpha diversity indices between the study groups.

|  | **Overall ‘p’ value** | **Intergroup ‘p’ value** | **HC**  **vs**  **T1DM** | **HC**  **vs T2DM** | **HC**  **vs T3DM** | **T1DM vs T2DM** | **T1DM vs T3cDM** | **T2DM vs T3cDM** |
| --- | --- | --- | --- | --- | --- | --- | --- | --- |
| **Chao 1** | <0.0001 | p value | 0.005 | 0.001 | 0.80 | 0.62 | 0.001 | <0.0001 |
|  |  | Adj. p value | **0.03** | **0.003** | 1.00 | 1.00 | **0.004** | **<0.0001** |
| **Evenness index** | 0.04 | p value | 0.04 | 0.46 | 0.68 | 0.16 | 0.01 | 0.19 |
|  |  | Adj. p value | 0.23 | 1.00 | 1.00 | 0.98 | **0.04** | 1.00 |
| **Shannon-H index** | 0.009 | p value | 0.26 | 0.002 | 0.50 | 0.06 | 0.52 | 0.004 |
|  |  | Adj. p value | 1.00 | **0.01** | 1.00 | 0.39 | 1.00 | **0.02** |
| **Fischer alpha** | <0.0001 | p value | 0.001 | 0.001 | 0.67 | 0.95 | <0.0001 | <0.0001 |
|  |  | Adj. p value | **0.01** | **0.003** | 1.00 | 1.00 | **<0.0001** | **<0.0001** |

**Footnote**: Bonferroni correction for multiple hypothesis testing was performed prior to deriving the overall p values. The intergroup p values are based on Tukey’s Post Hoc test. Adj. indicates adjusted.

**Supplementary figure 2:** Scree plot for PCA.

**
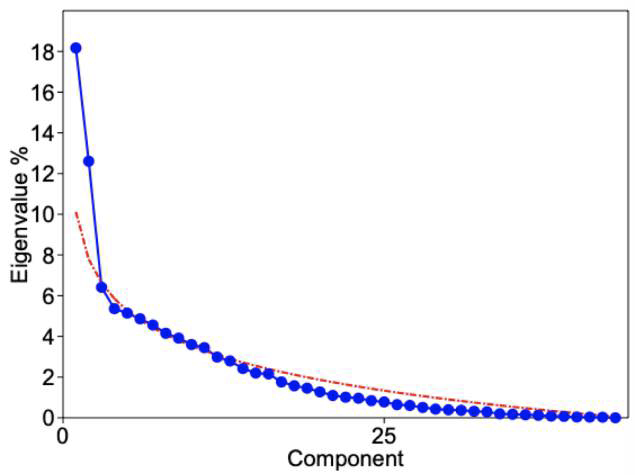
**

**Supplementary figure 3:** Clustering of species level OTUs between **(a)** healthy controls and all diabetic patients; and **(b)** between the three diabetic groups.


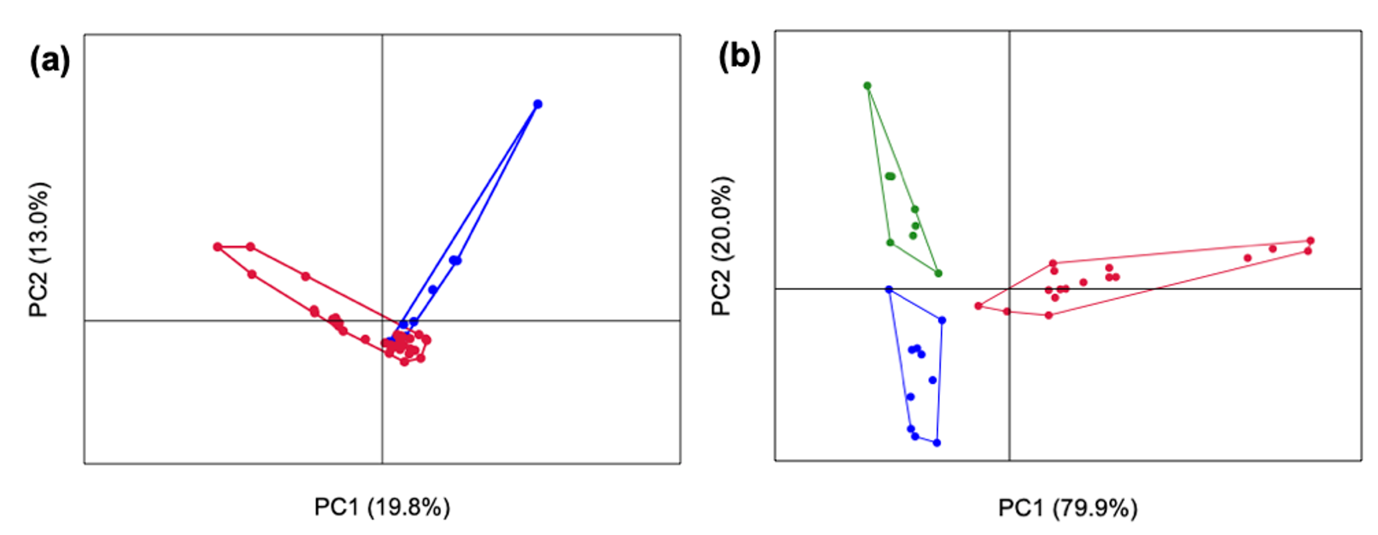


**Supplementary Figure 4:** Firmicutes to Bacteroidetes ratio in each of the study groups.


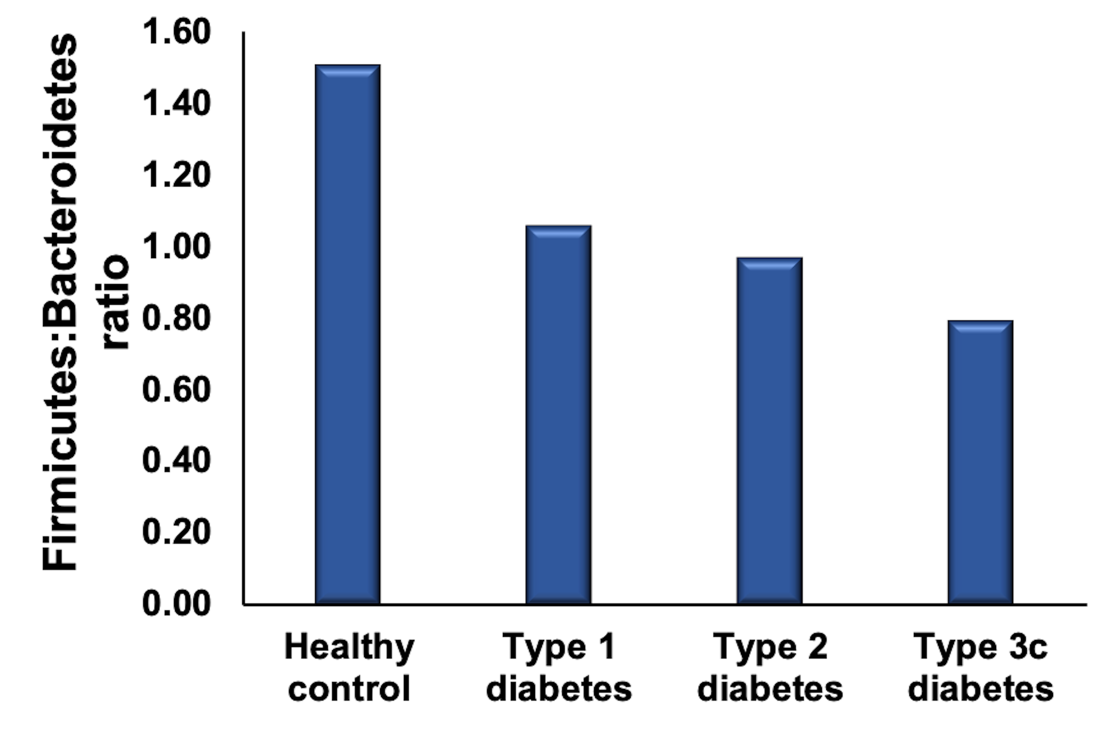


|  | **Overall ‘p’ value** | **Intergroup ‘p’ value** | **HC**  **vs**  **T1DM** | **HC**  **vs T2DM** | **HC**  **vs T3DM** | **T1DM vs T2DM** | **T1DM vs T3cDM** | **T2DM vs T3cDM** |
| --- | --- | --- | --- | --- | --- | --- | --- | --- |
| **Firmicutes** | <0.0001 | p value | 0.27 | 0.25 | <0.0001 | 0.99 | 0.005 | 0.003 |
|  |  | Adj. p value | 1.00 | 1.00 | **<0.0001** | 1.00 | **0.03** | **0.01** |
| **Bacteroidetes** | 0.04 | p value | 0.65 | 0.62 | 0.07 | 0.99 | 0.02 | 0.01 |
|  |  | Adj. p value | 1.00 | 1.00 | 0.44 | 1.00 | 0.15 | 0.09 |
| **Actinobacteria** | <0.0001 | p value | 0.19 | 0.56 | <0.0001 | 0.43 | 0.008 | <0.0001 |
|  |  | Adj. p value | 1.00 | 1.00 | **<0.0001** | 1.00 | 0.04 | **0.001** |
| **Proteobacteria** | <0.0001 | p value | 0.92 | 0.52 | 0.004 | 0.52 | 0.004 | <0.0001 |
|  |  | Adj. p value | 1.00 | 1.00 | **0.03** | 1.00 | **0.03** | **0.001** |

**Supplementary Table 4**: Significance levels of differences in the phylum level taxa between the study groups.

**Footnote**: Bonferroni correction for multiple hypothesis testing was performed prior to deriving the overall p values. The intergroup p values are based on Tukey’s Post Hoc test. Adj. indicates adjusted.

**Supplementary Figure 5:** Stacked bars showing the relative abundances of different levels of taxa for individual controls and patients. **(a)** Phylum; **(b)** Class; **(c)** Order; **(d)** Family.

**

**

**Supplementary Figure 6:** PCoA plots showing significantly different clustering of Type 3 diabetes compared to controls, Type 1 and Type2 diabetes in **(a)** Order; **(b)** Class; and **(c)** Family level taxa. Heatmaps depicting groupwise relative abundances of organisms at the **(d)** Order; **(e)** Class; and **(f)** Family level taxa.

**
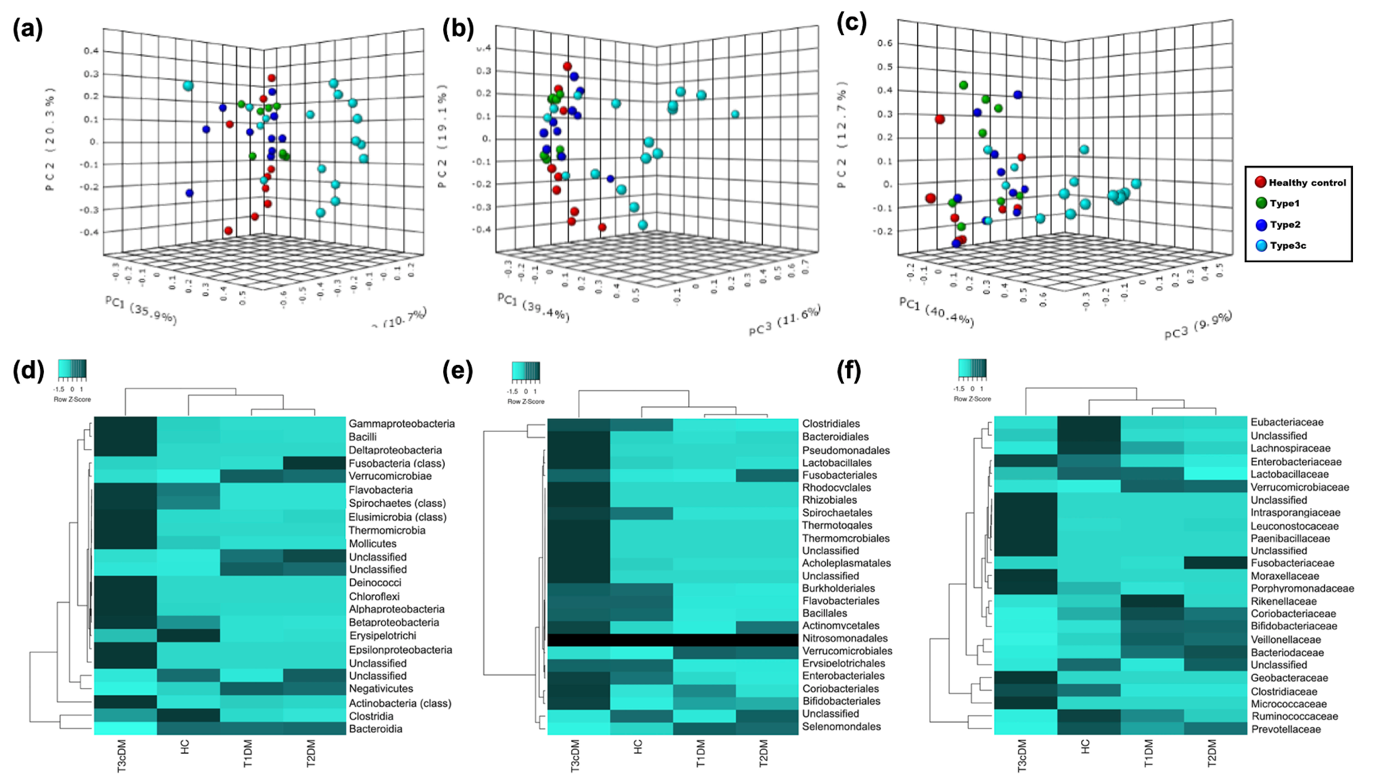
**

**Supplementary Table 5**: Significance levels of differences in relative abundances of genera between the study groups

|  | **Overall ‘p’ value** | **Intergroup ‘p’ value** | **HC**  **vs**  **T1DM** | **HC**  **vs T2DM** | **HC**  **vs T3DM** | **T1DM vs T2DM** | **T1DM vs T3cDM** | **T2DM vs T3cDM** |
| --- | --- | --- | --- | --- | --- | --- | --- | --- |
| ***Prevotella*** | **0.023** | p value | 0.32 | 0.70 | 0.007 | 0.53 | 0.14 | 0.02 |
|  |  | Adj. p value | 1.00 | 1.00 | **0.04** | 1.00 | 1.00 | 0.11 |
| ***Nesterenkonia*** | **<0.0001** | p value | 0.78 | 0.49 | 0.001 | 0.71 | 0.001 | <0.0001 |
|  |  | Adj. p value | 1.00 | 1.00 | **0.009** | 1.00 | **0.004** | **<0.0001** |
| ***Clostridium*** | **<0.0001** | p value | 0.04 | 0.01 | 0.04 | 0.78 | <0.0001 | <0.0001 |
|  |  | Adj. p value | 0.22 | 0.08 | 0.24 | 1.00 | **<0.0001** | **<0.0001** |
| ***Geobacter*** | **<0.0001** | p value | 1.00 | 1.00 | <0.0001 | 1,00 | <0.0001 | <0.0001 |
|  |  | Adj. p value | 1.00 | 1.00 | **<0.0001** | 1.00 | **<0.0001** | **<0.0001** |
| ***Acinetobacter*** | **0.003** | p value | 0.23 | 0.48 | 0.05 | 0.58 | 0.001 | 0.005 |
|  |  | Adj. p value | 1.00 | 1.00 | 0.32 | 1.00 | **0.007** | **0.03** |
| ***Porphyromonas*** | **<0.0001** | p value | 0.04 | 0.004 | 0.11 | 0.49 | <0.0001 | <0.0001 |
|  |  | Adj. p value | 0.22 | 0.02 | 0.63 | 1.00 | **0.001** | **<0.0001** |
| ***Veillonella*** | **0.002** | p value | 0.46 | 0.85 | 0.003 | 0.35 | 0.05 | 0.001 |
|  |  | Adj. p value | 1.00 | 1.00 | **0.02** | 1.00 | 0.28 | **0.01** |
| ***Fusobacterium*** | 0.40 | p value | na | na | na | na | na | na |
|  |  | Adj. p value | na | na | na | na | na | na |
| ***Parabacteroides*** | **0.001** | p value | 0.19 | 0.30 | 0.04 | 0.74 | 0.001 | 0.001 |
|  |  | Adj. p value | 1.00 | 1.00 | 0.23 | 1.00 | **0.003** | **0.005** |
| ***Akkermansia*** | 0.09 | p value | na | na | na | na | na | na |
|  |  | Adj. p value | na | na | na | na | na | na |
| ***Lactobacillus*** | **0.042** | p value | 0.77 | 0.21 | 0.03 | 0.13 | 0.02 | 0.41 |
|  |  | Adj. p value | 1.00 | 1.00 | 0.17 | 1.00 | 0.09 | 1.00 |
| ***Mitsuokella*** | **0.008** | p value | 0.65 | 0.48 | 0.003 | 0.82 | 0.02 | 0.02 |
|  |  | Adj. p value | 1.00 | 1.00 | **0.02** | 1.00 | 0.11 | 0.14 |
| ***Ruminococcus*** | **<0.0001** | p value | 0.81 | 0.79 | <0.0001 | 0.62 | 0.001 | <0.0001 |
|  |  | Adj. p value | 1.00 | 1.00 | **0.002** | 1.00 | **0.01** | **<0.0001** |
| ***Eubacterium*** | **<0.0001** | p value | 0.08 | 0.008 | <0.0001 | 0.46 | 0.005 | 0.03 |
|  |  | Adj. p value | 0.45 | 0.05 | **<0.0001** | 1.00 | **0.03** | 0.18 |
| ***Dialister*** | **0.004** | p value | 0.009 | 0.001 | 0.06 | 0.53 | 0.25 | 0.05 |
|  |  | Adj. p value | 0.05 | **0.004** | 0.34 | 1.00 | 1.00 | 0.28 |
|  |  |  |  |  |  |  |  |  |
|  | **Overall ‘p’ value** | **Intergroup ‘p’ value** | **HC**  **vs**  **T1DM** | **HC**  **vs T2DM** | **HC**  **vs T3DM** | **T1DM vs T2DM** | **T1DM vs T3cDM** | **T2DM vs T3cDM** |
| ***Unclassified from Clostridiales*** | **0.021** | p value | 0.11 | 0.13 | 0.002 | 0.88 | 0.24 | 0.15 |
|  |  | Adj. p value | 0.67 | 0.77 | **0.01** | 1.00 | 1.00 | 0.89 |
| ***Butyrivibrio*** | **<0.0001** | p value | 0.001 | <0.0001 | 0.15 | 0.80 | 0.02 | 0.006 |
|  |  | Adj. p value | **0.01** | **0.001** | 0.90 | 1.00 | 0.13 | **0.04** |
| ***Roseburia*** | **<0.0001** | p value | 0.77 | 0.73 | 0.001 | 0.52 | <0.0001 | 0.001 |
|  |  | Adj. p value | 1.00 | 1.00 | **0.003** | 1.00 | **0.001** | **0.008** |
| ***Bifidobacterium*** | **0.001** | p value | 0.02 | 0.59 | 0.13 | 0.07 | <0.0001 | 0.03 |
|  |  | Adj. p value | 0.14 | 1.00 | 0.76 | 0.43 | **<0.0001** | 0.17 |
| ***Collinsella*** | **0.001** | p value | 0.28 | 0.97 | 0.01 | 0.25 | <0.0001 | 0.01 |
|  |  | Adj. p value | 1.00 | 1.00 | 0.08 | 1.00 | **0.002** | 0.07 |
| ***Alistipes*** | **<0.0001** | p value | 0.22 | 0.35 | 0.005 | 0.03 | <0.0001 | 0.06 |
|  |  | Adj. p value | 1.00 | 1.00 | **0.03** | 1.00 | **<0.0001** | 0.37 |
| ***Bacteroides*** | **0.007** | p value | 0.13 | 0.87 | 0.07 | 0.09 | 0.001 | 0.09 |
|  |  | Adj. p value | 0.81 | 1.00 | 0.45 | 0.55 | **0.004** | 0.59 |
| ***Unclassified from Bacteria*** | **0.005** | p value | 0.12 | 0.90 | 0.003 | 0.15 | 0.27 | 0.004 |
|  |  | Adj. p value | 0.75 | 1.00 | **0.02** | 0.88 | 1.00 | **0.02** |
| ***Faecalibacterium*** | **<0.0001** | p value | 0.81 | 0.49 | 0.001 | 0.38 | <0.0001 | 0.007 |
|  |  | Adj. p value | 1.00 | 1.00 | **0.005** | 1.00 | **0.003** | **0.04** |
| ***Megasphaera*** | **0.001** | p value | 0.04 | 0.26 | 0.13 | 0.33 | <0.0001 | 0.005 |
|  |  | Adj. p value | 0.27 | 1.00 | 0.81 | 1.00 | **0.001** | **0.03** |

**Footnote**: Bonferroni correction for multiple hypothesis testing was performed prior to deriving the overall p values. The intergroup p values are based on Tukey’s Post Hoc test. Adj. indicates adjusted.

**Supplementary Figure 7:** Relative abundances of the top 25 species according to the study groups

.
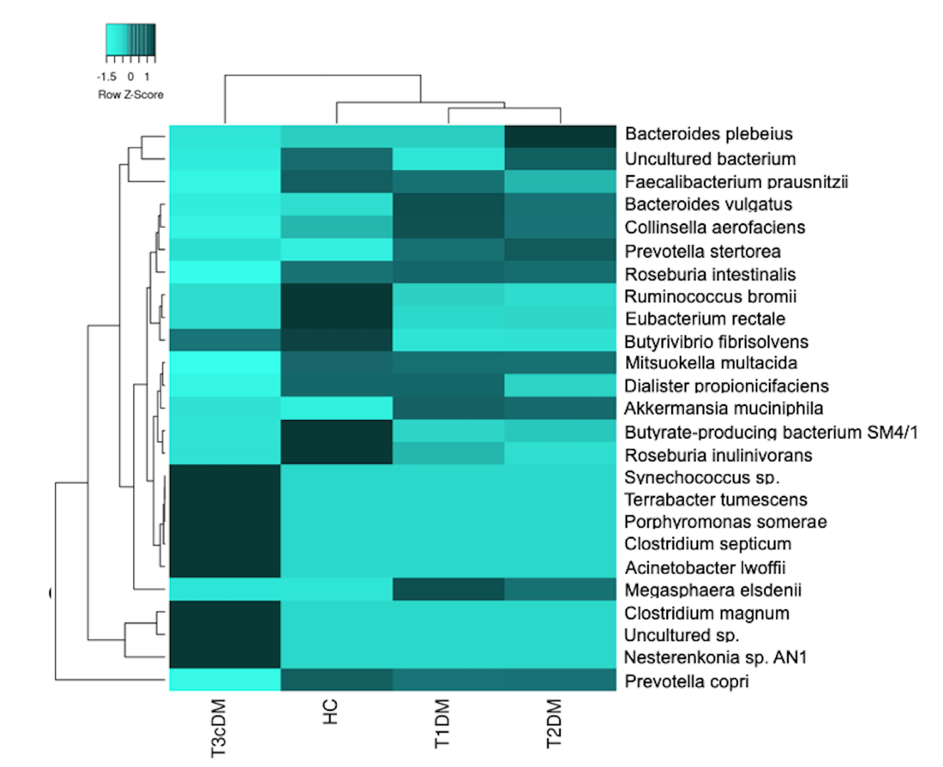


**Supplementary Table 6**: Significance levels of differences in relative abundances of species between the study groups

|  | **Overall ‘p’ value** | **Intergroup ‘p’ value** | **HC**  **vs**  **T1DM** | **HC**  **vs T2DM** | **HC**  **vs T3DM** | **T1DM vs T2DM** | **T1DM vs T3cDM** | **T2DM vs T3cDM** |
| --- | --- | --- | --- | --- | --- | --- | --- | --- |
| ***Prevotella copri*** | **<0.0001** | p value | 0.40 | 0.93 | <0.0001 | 0.44 | 0.008 | <0.0001 |
|  |  | Adj. p value | 1.00 | 1.00 | **0.001** | 1.00 | **0.04** | **0.001** |
| ***Nesterenkonia sp. AN1*** | **<0.0001** | p value | 0.71 | 1.00 | <0.0001 | 0.70 | 0.001 | <0.0001 |
|  |  | Adj. p value | 1.00 | 1.00 | **0.001** | 1.00 | **0.005** | **<0.0001** |
| ***Faecalibacterium praustnizii*** | **<0.0001** | p value | 0.91 | 0.49 | 0.001 | 0.44 | 0.001 | 0.006 |
|  |  | Adj. p value | 1.00 | 1.00 | **0.003** | 1.00 | **0.003** | **0.03** |
| ***Megasphaera elsdenii*** | **0.002** | p value | 0.06 | 0.35 | 0.13 | 0.30 | <0.0001 | 0.008 |
|  |  | Adj. p value | 0.35 | 1.00 | 0.76 | 1.00 | **0.02** | **0.04** |
| ***Clostridium septicum*** | **<0.0001** | p value | 0.93 | 0.75 | <0.0001 | 0.69 | <0.0001 | <0.0001 |
|  |  | Adj. p value | 1.00 | 1.00 | **0.001** | 1.00 | **0.002** | **<0.0001** |
| ***Butyrivibrio fibrisolvens*** | **<0.0001** | p value | 0.001 | <0.0001 | 0.13 | 0.69 | 0.02 | 0.003 |
|  |  | Adj. p value | **0.005** | **0.001** | 0.80 | 1.00 | 0.12 | **0.02** |
| ***Ruminococcus bromii*** | **<0.0001** | p value | 0.002 | <0.0001 | <0.0001 | 0.32 | 0.59 | 0.54 |
|  |  | Adj. p value | **0.01** | **<0.0001** | **<0.0001** | 1.00 | 1.00 | 1.00 |
| ***Collinsella aerofaciens*** | **0.001** | p value | 0.14 | 0.86 | 0.007 | 0.21 | <0.0001 | 0.002 |
|  |  | Adj. p value | 1.00 | 0.10 | **0.04** | 1.00 | **<0.0001** | **0.012** |
| ***Roseburia inulinivorans*** | **0.002** | p value | 0.29 | 0.06 | 0.001 | 0.48 | 0.03 | 0.15 |
|  |  | Adj. p value | 1.00 | 0.39 | **0.004** | 1.00 | 0.21 | 0.93 |
| ***Lactobacillus ruminis*** | **<0.0001** | p value | 0.23 | 0.82 | 0.002 | 0.47 | <0.0001 | 0.003 |
|  |  | Adj. p value | 1.00 | 1.00 | **0.009** | 1.00 | **<0.0001** | **0.015** |
| ***Bifidobacterium longum*** | **<0.0001** | p value | 0.18 | 0.91 | 0.004 | 0.21 | <0.0001 | 0.002 |
|  |  | Adj. p value | 1.00 | 1.00 | **0.025** | 1.00 | **<0.0001** | **0.012** |
| ***Butyrate producing bacterium SM4_1*** | **0.004** | p value | 0.79 | 0.46 | 0.002 | 0.66 | 0.008 | 0.02 |
|  |  | Adj. p value | 1.00 | 1.00 | **0.013** | 1.00 | 0.05 | 0.12 |
| ***Phorphyromonas somerae*** | **<0.0001** | p value | 0.49 | 0.47 | 0.004 | 1.00 | <0.0001 | <0.0001 |
|  |  | Adj. p value | 1.00 | 1.00 | **0.02** | 1.00 | **0.002** | **0.001** |
| ***Acinitobacter lwoffii*** | **<0.0001** | p value | 0.34 | 0.31 | 0.005 | 1.00 | <0.0001 | <0.0001 |
|  |  | Adj. p value | 1.00 | 1.00 | **0.03** | 1.00 | **0.001** | **<0.0001** |
| ***Clostridium magnum*** | **<0.0001** | p value | 0.51 | 0.49 | 0.002 | 1.00 | <0.0001 | <0.0001 |
|  |  | Adj. p value | 1.00 | 1.00 | **0.009** | 1.00 | **0.001** | **<0.0001** |

**Footnote**: Bonferroni correction for multiple hypothesis testing was performed prior to deriving the overall p values. The intergroup p values are based on Tukey’s Post Hoc test. Adj. indicates adjusted.

**Supplementary Figure 8:** Pre and post normalization values for peak areas of **(a)** amino acids, **(b)** fatty acids and **(c)** non-fatty organic acids. PCoA plots representing the clustering of **(d)** amino acids, **(e)** Fatty acids, and **(f)** Non-fatty organic acids.

PCoA plots were generated using Bray Curtis distance matrix.

**
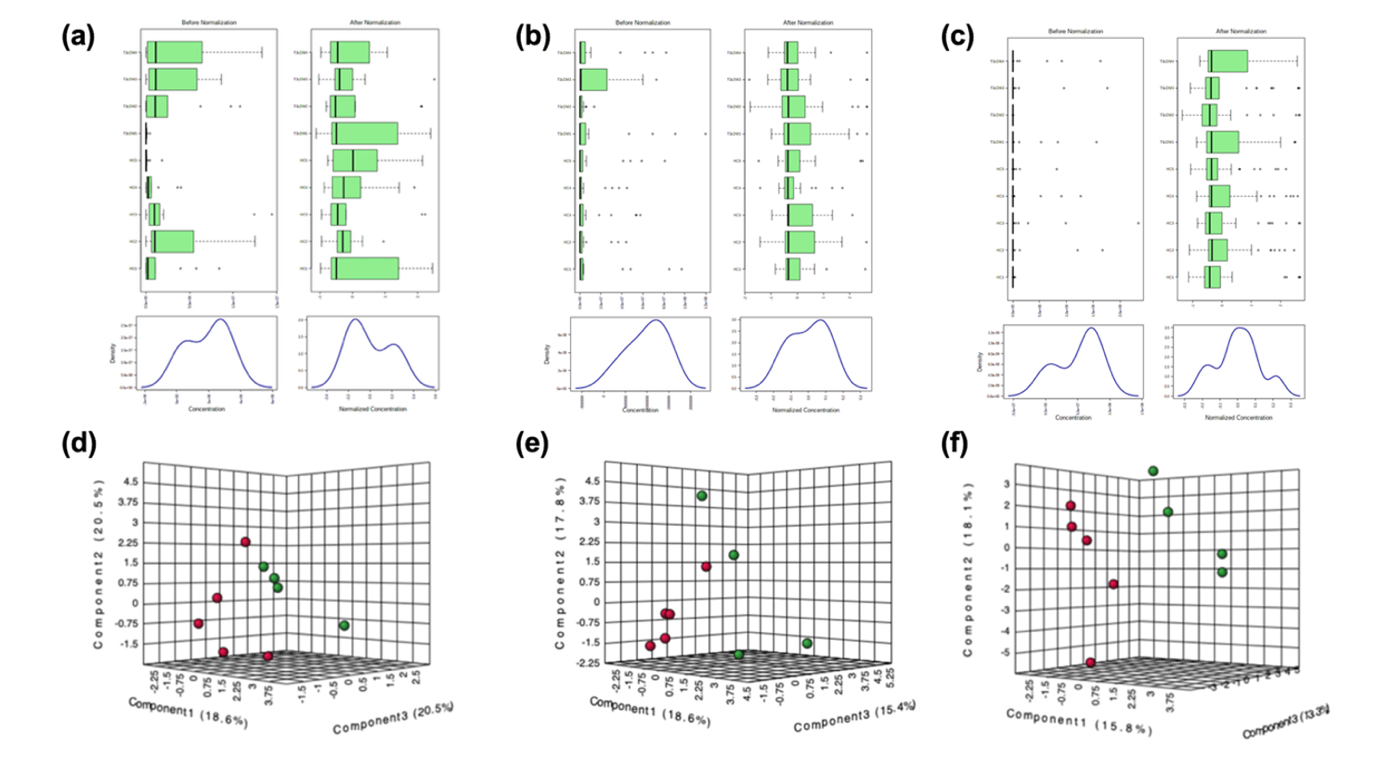
**

**Footnote:** The red dots in the PCoA plots indicate healthy controls while the green dots indicate Type 3c diabetes.

**Supplementary Figure 9:** Microbiome-metabolome networks depicting interactions of bacteria with **(a)** amino acids, **(b)** fatty acids, and **(c)** non-fatty organic acids.


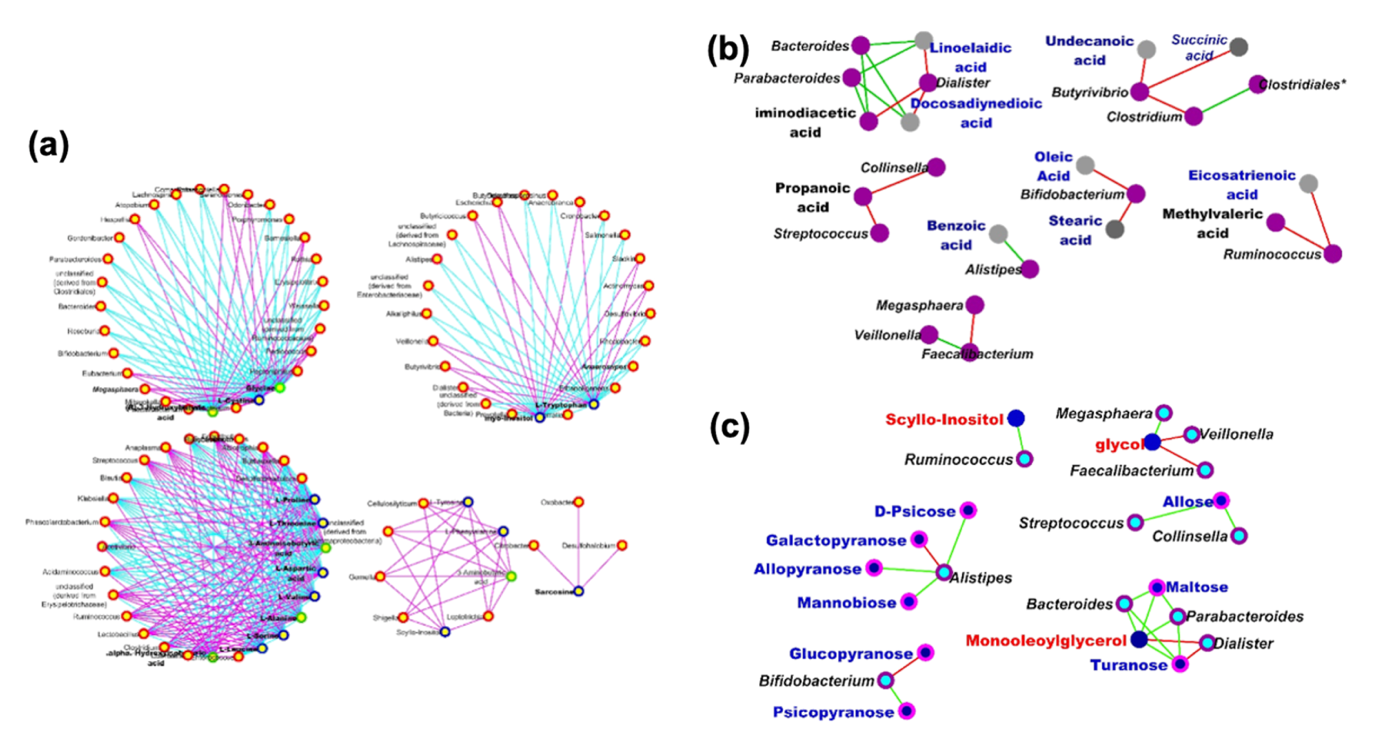

Supplement: Supplementary file 1 — Supplementary Information. [file 41598_2021_90024_MOESM1_ESM.docx]
